# Adapting Static and Contextual Representations for Policy Gradient-Based Summarization

**DOI:** 10.3390/s23094513

**Published:** 2023-05-05

**Authors:** Ching-Sheng Lin, Jung-Sing Jwo, Cheng-Hsiung Lee

**Affiliations:** 1 Master Program of Digital Innovation, Tunghai University, Taichung 40704, Taiwan; 2 Department of Computer Science, Tunghai University, Taichung 40704, Taiwan

**Keywords:** automatic text summarization, GloVe, BERT, GPT, unsupervised training, policy gradient reinforcement learning

## Abstract

Considering the ever-growing volume of electronic documents made available in our daily lives, the need for an efficient tool to capture their gist increases as well. Automatic text summarization, which is a process of shortening long text and extracting valuable information, has been of great interest for decades. Due to the difficulties of semantic understanding and the requirement of large training data, the development of this research field is still challenging and worth investigating. In this paper, we propose an automated text summarization approach with the adaptation of static and contextual representations based on an extractive approach to address the research gaps. To better obtain the semantic expression of the given text, we explore the combination of static embeddings from GloVe (Global Vectors) and the contextual embeddings from BERT (Bidirectional Encoder Representations from Transformer) and GPT (Generative Pre-trained Transformer) based models. In order to reduce human annotation costs, we employ policy gradient reinforcement learning to perform unsupervised training. We conduct empirical studies on the public dataset, Gigaword. The experimental results show that our approach achieves promising performance and is competitive with various state-of-the-art approaches.

## 1. Introduction

Automatic text summarization is a technique used to produce a concise version of a source document that preserves its essence while removing redundant information [1]. It is always an important research task in the natural language processing community. Due to the ever-growing volume of electronic documents made available in our daily lives, the need for an efficient summarization tool and reliable approach to save readers’ time is also increasing.

In general, automatic text summarization is categorized into extractive and abstractive summarization. The extraction-based approach usually composes a summary by selecting salient parts from the source text and concatenating them to create the final results [2], while the abstractive-based approach often requires rewriting and synthesizing the text content in order to produce a summary, instead of directly extracting the crucial text from the source document [3]. An example comparison can be found in Table 1. The input document is taken from a CNN news article, where the extractive summarization identifies important sentences by applying BERT (Bidirectional Encoder Representations from Transformer) embeddings and performing K-Means clustering [4], and the abstractive summarization trains a sequence-to-sequence model for the gap-sentences generation task [5].

How to represent the text in a numerical form for the machine to process is an important step of NLP pipelines and also has significant impact on the summarization performance [6]. Although the traditional bag-of-words (BOW) model is simple and commonly used, it is not able to properly capture semantic relationships when two sentences have no words in common but share similar semantics [7]. For example, consider the following two sentences: “Brian left Beach Boys” and “Wilson abandoned the band”; they do not share any common words but have the same semantics. Most recently, word embeddings have gained much attention because of their ability to convert words into low-dimensional and dense representations with the aid of neural network language modeling. Global Vectors (GloVe) are an unsupervised method used to learn word representations by computing word–word co-occurrence, and the model is trained on Wikipedia and Gigaword corpora [8]. BERT (Bidirectional Encoder Representations from Transformers) [9] is a pre-trained language model based on the Transformer architecture [10] for the purpose of learning the semantic information of the input text. It is trained on two learning objectives, a Masked Language Model (MLM) and Next Sentence Prediction (NSP). BERT has been widely adopted in NLP models and has achieved state-of-the-art results on 11 NLP tasks. GPT-2 (Generative Pre-trained Transformer 2) is also a pre-trained language model but based on the decoder of the Transformer architecture [11]. Unlike BERT, which learns the words in a bidirectional manner, the training of GPT-2 is conducted in an autoregressive fashion by the task of predicting the next word. The word embeddings of GloVe are static, where each word has a fixed vector representation. On the other hand, BERT and GPT-2 produce contextualized representations where the word embeddings are context-sensitive [12].

When learning text summarization with supervised training methods, the training data are usually not available, and it is often difficult to annotate on a large scale. Unsupervised learning is becoming popular because it is more applicable in real-world practice [13]. Reinforcement learning (RL)-based summarization models that maximize a reward function [14] and adversarial training methods for text generation [15] are two mainstream approaches to unsupervised extractive summarization. There have also been some efforts on automatically generating training data [16] and better learning text representations in a self-supervised fashion [17].

In this paper, we employ a policy gradient reinforcement learning model for unsupervised extractive summarization. Since text generation in the neural network model often suffers from a non-differentiable issue, reinforcement learning methods devise rewards to overcome this challenge [18]. Moreover, to better encode the semantics of words, we investigate the effectiveness of adapting and combing various embedding schemes to represent words. We formulate the extraction of important tokens from the given text as a sequence labeling task with binary labels to find an optimal assignment. The mechanism of our method is analogous to the Generative Adversarial Networks (GAN) [19], where the RL agent plays the role of ‘generator’ for performing binary sequence labeling and the reward function plays the role of ‘discriminator’ for shaping the agent’s behavior. The major difference is that our reward functions do not accept any real samples and are not trainable networks.

The primary contributions of this paper are two-fold:

- We propose an automated model for unsupervised extractive text summarization based on a policy gradient reinforcement learning approach. The semantic representations of the text are extracted from static and contextual embeddings, including GloVe-, BERT- and GPT-based models.
- Empirical studies on the Gigaword dataset illustrate that the proposed solution is capable of creating reasonable summaries and is comparable with other state-of-the-art algorithms.

The rest of this paper is organized in the following manner. In Section 2, several research efforts and techniques related to the topic of this paper are reviewed. We then discuss the proposed approach and detailed components in Section 3. Experimental studies are reported to justify the effectiveness and analyze the performance of the proposed method in Section 4. Finally, we draw some conclusions and also provide several possible future research paths.

## 2. Related Work

Automatic text summarization methods which support a digest of a source document are classified into two categories: one is extractive summarization and the other is abstractive summarization [20]. Extraction-based approaches produce summaries by selecting crucial parts of the original document and combining them to derive a reasonable summary. On the other hand, abstraction-based methods rely on understanding the gist of the source document to rewrite a condensed version. A Restricted Boltzmann Machine (RBM) approach is used to extract features for extractive text summarization [21]. BERT [9], a pre-trained self-supervised language model, has successfully outperformed many state-of-the-art algorithms for natural language processing-related tasks. BertSum is a BERT-based model to learn sentence representation and give each sentence a score indicating whether the sentence should be extracted as a candidate summary sentence [22]. The combination of sequence-to-sequence learning (seq2seq) and an attention mechanism is a widely used approach that compresses the input into a vector representation with an encoder and generates the output with a decoder [23]. Many different variants of attention-based seq2seq networks have been explored for abstractive summarization tasks [3,24]. A GAN is an adversarial training framework where two networks, a Generator and a Discriminator, are trained alternately in a competitive fashion [19]. It has been proposed to build a Generator to produce the abstractive summarization based on the raw input text, whereas learning is performed by a Discriminator to distinguish between the generated summary and a human-written summary [18].

A two-stage design has recently been attempted for constructing document summarization systems. EXCONSUMM proposes a hybrid extract-then-compress model to avoid the challenges in abstractive summarization but still yield more readable summaries than extraction-based models [25]. Since human-written summary sentences are usually generated from a single sentence or the fusion of multiple sentences, a joint-score method to capture the semantic representation for both singletons and sentence pairs is proposed [26]. Instances with a higher score are then extracted and rewritten with the leverage of the Pointer-Generator Network [27].

RL aims at training an agent to interact with an environment for the purpose of generating a feasible policy which could perform a proper action in a given state in order to maximize rewards [28]. It has been broadly and increasingly adopted in many applications such as autonomous driving, robotics and video games. The research area of text summarization is of no exception, where many related approaches have been proposed and have yielded state-of-the-art results [29]. An extractive technique based on a graph and reinforcement neural network, GoSum, is proposed to build a heterogeneous graph for the source document and then apply a policy gradient to optimize the reward scores for the summarization of long papers [30]. As coherence is the key factor to produce a high-quality summary, efforts to develop learning models which could take both in-sentence dependencies and cross-sentence semantics into consideration are necessary. The Reinforced Neural Extractive Summarization model [31] learns a neural extractive summarizer based on the REINFORCE algorithm, where the reward score is mainly provided by another neural model, ARC-II, which is used to measure the degree of coherence between sentences [32]. Unlike traditional abstractive summarization approaches which assume a deterministic target distribution and are trained by maximum likelihood estimation, the BRIO model applies non-deterministic distribution to assign probability mass for candidate summaries based on their quality [33]. To imitate the human procedure when writing a summary, FusionSum [34] devises a module to combine salient sentences into different groups and another module to rewrite the summary sentences for each group. The training mechanism between the two modules is based on an end-to-end cooperative reinforcement learning.

Based on summarization approaches, extractive methods only extract information from the original text, which could be more factually accurate but may also introduce redundant or uninformative text. Abstractive approaches can produce more concise and readable summaries, but the text generated by the model may contain errors or inaccuracies. Based on learning methods, supervised summarizers can achieve more reliable results but need training data with human input. Unsupervised summarizers do not require labelled data but could result in summaries that are difficult to understand.

## 3. Proposed Method

**Figure 1:**
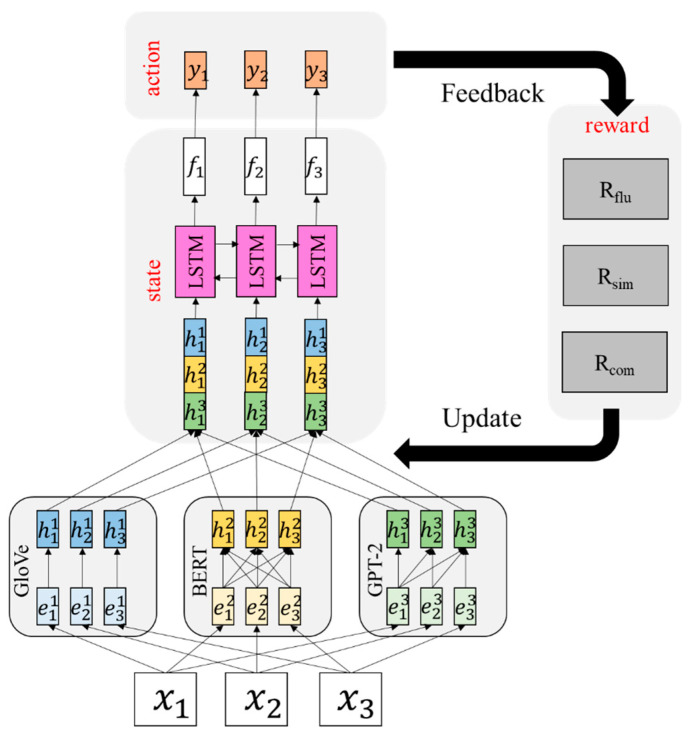
The architecture of the proposed policy gradient reinforcement learning model for unsupervised extractive summarization.

In this section, we will present the proposed network, which contains a policy gradient reinforcement learning architecture, representation learning with various embeddings and optimization strategies (Figure 1).

### 3.1. The Policy Gradient Reinforcement Learning Architecture

In this work, we formulate the task of extractive summarization as the selection of important tokens from the document and solve the problem with a reinforcement learning algorithm. The input of the algorithm is a document $d_{i}$ with t characters (i.e., $\left(x_{1},{\;x}_{2},\ldots x_{t}\right)$), and the goal is to predict a binary label for each character $x_{j}$ to determine whether to include $x_{j}$ in the final summary. The output will be the concatenation of those chosen items.

Reinforcement learning is a popular model to solve planning problems and has been widely used to learn in highly dynamic and complex environments. The learning agent, in general, follows three steps repeatedly with a trial-and-error mechanism to learn the optimal policy. First, the agent observes the environment state ***s***. Second, based on a policy, the agent takes an action ***a*** from the given state ***s***. Third, the agent receives a reward ***r*** for taking action ***a*** in state ***s*** and the environment will subsequently provide a next state ***s’***. Ultimately, the general goal of the agent is to maximize the accumulated rewards.

We describe how to incorporate our summarization approach to the reinforcement learning settings as follows:

- State: A document $d_{i}=\left(x_{1},{\;x}_{2},\ldots x_{t}\right)$ is considered as a state ***s***. Each token $x_{j}$ is further represented by the concatenation of its GloVe ($h^{1}$), BERT($h^{2}$) and GPT-2($h^{3}$) embeddings. GloVes are a kind of static embeddings, the BERT is a category of dynamic embeddings trained on masked language modeling and GPT-2 is another type of dynamic embeddings trained on autoregressive language modeling. We then pass the concatenated embeddings into an LSTM layer to encode the sequential information and capture contextual features. The state representation is then denoted as f [35,36].
- Action: The selection of important words from a sentence is treated as a sequence labelling problem in this work. Given the state representation f, the algorithm will perform the action of producing a sequence of binary labels $y=\left(y_{1},{\;y}_{2},\ldots y_{t}\right)$ to indicate the importance of a word.
- Reward: We apply three commonly used reward functions to measure the quality of the extracted text, including fluency ($R_{flu}$), similarity ($R_{sim}$) and compression ($R_{com}$) [14,36,37]. The fluency reward judges if the generated text is grammatically sound and semantically correct. Its score is calculated as the average of their perplexities by a language model. The similarity reward measures the semantic similarity between the generated summary and the source document in order to ensure the content’s preservation. We adopt the cosine similarity as the similarity score to compute the distance between the embeddings of the generated summary and the source document. The compression reward encourages the agent to generate summaries close to the predefined length. We refer to the prior research work [14], $R_{com}\left(\left|y\right|,L\right)=\exp\left(-\frac{\left|\left|y\right|-L\right|}{\sigma_{L}}\right)$, to calculate the compression score, where $\left|y\right|$ is the length of the generated summary, L is the target summary length and $\sigma_{L}$ is a hyper-parameter.

### 3.2. Training Algorithm

In this work, we use a policy gradient method to learn the summarization task. The learnable policy is implemented in a neural network structure, which is parameterized by θ. Given an input document ($d_{i}$), the state representation f is obtained after concatenating the embeddings of GloVe, BERT and GPT-2 and subsequently passing them through the LSTM layer. The policy is defined as multiplication of the probability of the token $y_{i}$ being included into the summary:

(1) $$\pi_{\theta }(y|f)=\underset{i}{\prod }\mathrm{Pr}(y_{i}|f,\;\theta )$$

After sampling the action based on $\pi_{\theta }$, the reward $r^{\pi }\;$will be given to evaluate the selected tokens. The goal of the learning is to maximize the reward, which is denoted as $J\left(\theta \right)$, and the gradient can be derived as shown in Equation (2) by applying a policy gradient theorem [38]:

(2) $$\nabla_{\theta }J\left(\theta \right)=\nabla_{\theta }\left(r^{\pi }\right){\log\pi }_{\theta }(y|f)$$

We use the gradient to update the weight of θ with the learning rate α as follows:

(3) $$\theta \leftarrow \theta +\alpha \nabla_{\theta }J\left(\theta \right)$$

Algorithm 1 shows the overall algorithm to train our proposed model.
**Algorithm 1:** Policy Gradient-Based Summarization Model**Parameters**:  θ for the policy network π.**for** each training iteration:$\;\mathrm{Sample}\;m\;\mathrm{examples}\;\mathrm{of}\;\mathrm{input}\;\mathrm{document}\;\left\{d_{1},{\;d}_{2},\ldots d_{m}\right\}$$\;\mathrm{for}\;\mathrm{each}\;d_{i}$ $\mathrm{with}\;t\;\mathrm{characters}\;\left\{x_{1},{\;x}_{2},\ldots x_{t}\right\}$ as a state s $\;\;\mathrm{Convert}\;\left\{x_{1},{\;x}_{2},\ldots x_{t}\right\}$ to $\left\{f_{1},{\;f}_{2},\ldots f_{t}\right\}$$\;\;\mathrm{Perform}\;\mathrm{an}\;\mathrm{action}\;\mathrm{by}\;\mathrm{producing}\;a\;\mathrm{binary}\;\mathrm{probability}\;\mathrm{distribution}\;\mathrm{based}\;\mathrm{on}\;\mathrm{Equation}\;(1)\;\mathrm{for}\;\mathrm{each}\;f_{j}$$\;\mathrm{to}\;\mathrm{obtain}\;\left\{y_{1},{\;y}_{2},\ldots y_{t}\right\}$   Calculate the rewards $R_{flu}$$,\;R_{sim}$ $\mathrm{and}\;R_{com}$   Compute the gradient in Equation (2)   Update θ in Equation (3) End for**end for**

## 4. Experiments and Results

In this section, we present empirical studies to illustrate our method with static and contextual embeddings for the summary generation. These studies consist of (1) the introduction of the dataset; (2) the definition of evaluation indices; (3) comparisons with other published approaches; and (4) ablation studies to analyze the effect of each embedding scheme.

### 4.1. Dataset

We apply our proposed framework on a public dataset, Gigaword. The training, validation and testing set sizes are 1 M, 189 K and 1951, respectively [24]. The annotation data are stored in JSON format, where each instance contains the ID, text and the corresponding summary. The average length of the source and summary are shown in Table 2.

### 4.2. Evaluation Metric

To automatically measure the summarization performance of the proposed network, Recall-Oriented Understudy for Gisting Evaluation (ROUGE), which is the most broadly used evaluation measure in the relevant research, is employed [39]. ROUGE measures the co-occurrence information between the machine-generated summary and ground truth summary. In general, there are three popular ROUGE variants for the summarization task, ROUGE-1 (R-1), ROUGE-2 (R-2) and ROUGE-L (R-L). R-N computes the similarity score based on the percentage of overlap in the N-gram, R-1 compares the overlap in the unigram and R-2 measures the overlap in the bigram. Meanwhile, R-L refers to the length ratio of the longest common sequence between the generated and ground-truth summaries to indicate fluency.

### 4.3. Experimental Results

In order to validate the effectiveness of our method, we conduct an experimental investigation to compare its results against those of other methods, which are introduced as follows:

- Lead-8: This approach is a simple baseline which directly selects the first eight words in the source document to assemble a summary.
- Contextual Match [40]: This research work introduces two language models to create the summarization and maintain output fluency. A generic pre-trained language model is used to perform contextual matching and the other target domain-specific language model is used to guide the generation fluency.
- AdvREGAN [15]: The method uses cycle-consistency to encode the input text representation and applies an adversarial reinforcement-based GAN to generate human-like text.
- HC_title_8 [37]: This work extracts words from the input text based on a hill-climbing approach by discrete optimization algorithms with a summary length of about eight words.
- HC_title_10 [37]: The model is identical to the HC_title_8 but with a summary length of about 10 words.
- SCRL_L8 [36]: The approach models the sentence compression to fine-tune BERT using a reinforcement learning setup.

The operating environment of this experimental comparison is the Windows 10 OS equipped with an Intel Core i9 central processing unit (CPU) and 128 GB of memory. The graphic processing unit (GPU) is an NVIDIA GeForce RTX 3090 with 24 GB of memory. Our hyper-parameter setting is displayed in Table 3.

Based on the comparison results shown in Table 4 where other baselines are directly taken from the corresponding papers, we obtain the best results on R-1 (29.75%) and R-L scores (26.98%), indicating their ability to produce informative and coherent summaries. Although our model is ranked second best on R-2 (10.26%), the value is very close to the best method (10.27%). Reinforcement learning-based approaches (SCRL_L8 and our model) with multiple reward functions yield better performance on R-1 and R-2.

In addition, we also provide several examples to illustrate our results in Table 5, including the input (INPUT_X), ground-truth summary (GOLD SUMMARY) and system-generated summary (GEN SUMMARY). As presented, our algorithm has the ability to extract crucial items and produce fair summaries. Nevertheless, there are still some mistakes to overcome for further improvements. First, because of partial selection from the source sentence to form the summary, the output is not always as grammatical as it should be (displayed in red text). Devising another reword function to incorporate grammatical constraints as the background knowledge could be a possible way to ensure grammaticality. Second, in some cases, the model fails to generate factually consistent summaries (displayed in blue text), reducing the faithfulness of the output and causing misunderstanding for the readers. One potential avenue for future research relates to leveraging textual entailment models to enhance factual consistency [41].

### 4.4. Ablation Experiments

In order to verify the feasibility of our proposals, we conduct ablation studies on the Gigaword dataset to investigate the benefits of our approach and quantify the effect of each embedding scheme. As the results show in Table 6, adapting static and contextual embeddings in our proposed method can exceed the results of training with GloVe, BERT and GPT-2 independently in terms of R-1, R-2 and R-3. Therefore, we believe that our method can further enhance the performance of producing summaries through learning complex features among different embedding schemes.

## 5. Conclusions

In this paper, we propose an unsupervised policy gradient reinforcement method based on the combination of various embeddings to resolve the text summarization problem. We conduct empirical studies on the Gigaword dataset, and the results are satisfactory. Compared to other existing methods, our model captures better representations of each token and yields the best performance in terms of ROUGE-1 and ROUGE-L.

Since the ROUGE-2 score of our method is ranked second (10.26%) and is only 0.01% lower than the top-performing method, future directions will focus on designing reward functions to increase bigram overlap. As our approach is capable of selecting important tokens, the investigation of combining chosen tokens into comprehensible phrases could be a possible solution.

Another challenge of our work will be the practical application in resource-constrained environments such as mobile computing due to the large embedding size. We are interested in knowledge distillation and low-dimensional representation for the purpose of deploying the proposed model in resource-restricted settings.

## Figures and Tables

**Table 1:** A CNN news article (https://edition.cnn.com/2015/04/14/americas/chile-same-sex-civil-unions/index.html) (accessed on 1 February 2023) is used to demonstrate the extractive and abstractive summarization.

**DOCUEMNT:** The country joined several of its South American neighbors in allowing the unions when President Michelle Bachelet enacted a new law on Monday. This is a concrete step in the drive to end the difference between homosexual and heterosexual couples, Bachelet said. The new law will take effect in six months. It will give legal weight to cohabiting relationships between two people of the same sex and between a man and a woman. The Chilean government estimates that around 2 million people will be able to benefit from the change. The law is intended to end discrimination faced by common-law couples, such as not being allowed to visit partners in hospital, make medical decisions on their behalf or decide what to do with their remains. It also gives the couples greater rights in the realms of property, health care, pensions and inheritance. A number of South American nations have moved to allow same-sex civil unions in recent years. But marriage between people of the same sex is legal only in Argentina, Brazil and Uruguay.
**EXTRACTIVE SUMMARY [4]:** The law is intended to end discrimination faced by common-law couples, such as not being allowed to visit partners in hospital, make medical decisions on their behalf or decide what to do with their remains.
**ABSTRACTIVE SUMMARY [5]:** Chile has become the first country in the world to legalise same-sex civil unions.

**Table 2:** Data statistics for the Gigaword dataset, where AvgInputLen is the average length of the input document and AvgSummaryLen is the average summary length.

	Training	Validation	Testing
AvgInputLen	31.19	31.32	29.70
AvgSummaryLen	8.04	8.31	8.79

**Table 3:** Hyper-parameter configuration.

Parameter	Value
Batch size	4
GloVe embeddings size	300
BERT embeddings size	768
GPT-2 embeddings size	768
LSTM hidden units	300
Learning rate	0.00005
Dropout rate	0.5

**Table 4:** Performance comparison (%).

	R-1	R-2	R-L
Lead-8	21.39	7.42	20.03
Contextual Match	26.48	10.05	24.41
AdvREGAN	27.29	10.01	24.59
HC_title_8	26.32	9.63	24.19
HC_title_10	27.52	**10.27**	24.91
SCRL_L8	29.64	9.98	26.57
Our Model	**29.75**	10.26	**26.98**

**Table 5:** Four example outputs produced by our model.

**INPUT_1:** un under-secretary-general for political affairs ibrahim gambari said on wednesday that although the future of the peace process in the middle east is hopeful, it still faces immense challenges.
**GOLD SUMMARY:** un senior official says peace process in middle east hopeful with immense challenges
**GEN SUMMARY:** under-secretary-general ibrahim gambari said future peace process still faces immense challenges
**INPUT_2:** rose UNK, a soprano who performed ## seasons at the metropolitan opera and established herself as a premier voice in american opera, has died.
**GOLD SUMMARY:** rose UNK metropolitan opera star in the ####s and ##s dies at ##
**GEN SUMMARY:** rose UNK soprano who performed at the opera has died.
**INPUT_3:** an over-loaded minibus overturned in a county in southwestern guizhou province tuesday afternoon, killing ## passengers and injuring three others.
**GOLD SUMMARY:** traffic accident kills ## injures # in sw china
**GEN SUMMARY:** over-loaded minibus in county in southwestern guizhou killing passengers others.
**INPUT_4:** south african cricket captain graeme smith said on sunday he would not use politics as an excuse for south africa ‘s performance in the cricket world cup in the west indies.
**GOLD SUMMARY:** cricket: politics not to blame for world cup failure: smith
**GEN SUMMARY:** african captain smith said politics an excuse in cricket cup

**Table 6:** Ablation experiment results (%).

	Summary		
	**R-1**	**R-2**	**R-L**
Our Model	**29.75**	**10.26**	**26.98**
GloVe	11.98	1.03	11.37
BERT	28.19	8.62	25.64
GPT-2	20.54	5.31	19.02

## Data Availability

Not applicable.
